# Very low calorie diet without aspartame in obese subjects: improved metabolic control after 4 weeks treatment

**DOI:** 10.1186/1475-2891-13-77

**Published:** 2014-07-28

**Authors:** Erik Norén, Henrik Forssell

**Affiliations:** 1 Department of General Surgery, Blekinge County Hospital, Lasarettsvägen, 371 85 Karlskrona, Sweden; 2 Blekinge Centre of Competence, Vårdskolevägen, 37181 Karlskrona, Sweden

**Keywords:** Obesity, VLCD, Fructose, Aspartame, Artificial sweetener, Weight reduction

## Abstract

**Background:**

Very low calorie diet (VLCD) is routinely used in programs for treatment of obesity and before bariatric surgery in order to reduce risk of postoperative complications. Aspartame, an artificial sweetener, is commonly used in VLCD and is well approved as a food additive without any adverse effects. The development of a new fructose containing VLCD formula without aspartame raises questions as to effects on glucose and lipid control.

**Methods:**

As part of an ongoing study of a novel bariatric surgery procedure, twenty-five obese subjects with mean body mass index (BMI) 39.8 kg/m^2^ and mean age of 48.8 years enrolled in a single center observational study. Seven subjects presented with type 2 diabetes mellitus. The subjects underwent four weeks dietary treatment with VLCD Slanka (Slanka®). Blood samples including fasting plasma glucose, HbA1c, cholesterol and triglycerides were performed at start and after four weeks of diet. Blood pressure and weight were noted.

**Results:**

All subjects completed the diet without any adverse events. Mean weight reduction was 8.2 kg with 95% confidence interval 7.1–9.2 kg (p = 0.001). Excess weight (i.e. proportion of weight exceeding BMI 25) loss decreased by median 19.5% (inter quartile range (IQR) 16,8-24,2). Median fasting plasma glucose was at inclusion 5,6 mmol/l (IQR 5,3-6,8) and after diet 4.8 mmol/l (IQR 4,6-5,2) (p = 0.001). Median HbA1c changed from 39 mmol/mol (IQR 37–44) to 37 mmol/mol (IQR 35–43) (p = 0.001). There was also significant reduction in cholesterol and triglyceride levels as well as in systolic blood pressure. Changes in other monitored blood chemistry values were without clinical importance.

**Conclusion:**

Four weeks treatment with fructose containing VLCD of obese subjects preparing for bariatric surgery gave a substantial weight reduction without any significant negative metabolic effects.

## Background

Adult obesity is defined by WHO as body mass index (BMI) greater than 30 kg/m^2^. Prevalence of obesity has been increasing worldwide over the last few decades. Obesity prevalence in the United States was 35.5% among men and 35.8% among women in 2009–2010, with no significant change compared to 2003–2008 [1]. In young and middle aged men and women all-cause mortality risk appear to be directly related to increase in BMI [2]. Bariatric surgery for severe obesity is associated with long-term weight loss and decreased overall mortality [3], in both sexes [4].

Very low calorie diet (VLCD) contains less than 800 kcal per day. VLCD are routinely being used prior to bariatric surgery. Such practice reduces liver volume [5], reduces postoperative complication rates and reduces the perceived technical difficulty of the procedure [6].

Aspartame, an artificial sweetener, is commonly used in VLCD and is well approved as a food additive without any adverse effects [7]. This has also been proven for heterozygotes for phenylketonuria (PKU) [8]. The development of a new VLCD formula (Slanka®) with fructose instead of aspartame raises questions as to effects on glucose and lipid control.

The total amount of carbohydrates in the Slanka formula is 37 g per 100 g. The daily amount of formula consumed during diet is approximately 180 g of which fructose is approximately 20 g. According to a review of health implications of fructose consumption [9], moderate fructose consumption of less than 50 g/day probably does not have any deleterious effect on lipid and glucose control. It is also known that diet rich in carbohydrates may have an increased risk of metabolic side effects [10].

The aim of this observational study is to investigate the effects of fructose containing VLCD in obese subjects, prior to bariatric intervention, on selected metabolic variables.

## Methods

As part of our ongoing single-center prospective study of a novel bariatric surgery procedure (Forssell H, Norén E, Six Month Results with Aspire Assist® - a Novel Endoscopic Weight Loss Therapy, submitted manuscript), twenty-five obese subjects consecutive enrolled after consent from the Regional Ethical Review Board in Lund, Sweden.

Baseline demographics are presented in Table 1. In summary there were 23 women and 2 men with mean BMI 39.8 kg/m^2^ and mean age 48.8 years. Seven subjects presented with type 2 diabetes mellitus (T2DM), four were treated with metformin, one with insulin and two had only dietary treatment. Other significant coexisting conditions are depicted in Table 2.

**Table 1:** Baseline demographics

	***Mean***	***Standard error***	***Range (min-max)***
Age (years)	48.8	1.66	33-65
BMI (kg/m^2^)	39.8	0.9	35.1-49
Weight (kg)	107.4	3.7	85.8-148
Excess weight (kg)	40.2	2.8	24.8-68.6

Baseline demographics at inclusion. Excess weight is weight exceeding BMI 25.

**Table 2:** Coexisting conditions in enrolled 25 subjects

***Condition under treatment***	***Number of subjects***
Type 2 diabetes mellitus (total)	7
- Dietary treatment	2
- Metformin	4
- Metformin + insulin	1
Hypertension (total)	8
- Diuretic	7
- ACE-inhibitor	4
- Beta receptor antagonist	3
Hyperlipidemia (total)	4
- Statin	4
Mood disorder (total)	7
- SSRI	7

The subjects underwent four weeks dietary treatment with VLCD Slanka®, Table 3*,* (Slanka Sverige AB, Sweden) corresponding to approximately 680 kcal per day (four portions). Slanka contains milk proteins and approximately 25 g lactose per 100 g formula. The main sweetener is sugar beet fructose and secondary Acesulfame K, an artificial sweetener that is excreted unchanged in urine. Slanka contains rapeseed oil fat (information from Slanka Sverige AB, Sweden).

**Table 3:** Slanka contents per portion and daily intake

	**Per portion 45 g**	**Daily intake 180 g**
Energy kcal/kJ	170/700	680/2800
Proteins g	17	68
Carbohydrate g (including Fructose g)	17 (3,5-5)	68 (14–20)
Fat g	4	16
Fibers g	1	4
Vitamin A mg	0.4	1.6
Vitamin D μg	1.8	7.2
Vitamin E mg	3.6	14.4
Vitamin K μg	23	92
Tiamine mg	0.6	2.4
Riboflavine mg	0.9	3.6
Niacine mg	6,2	24.8
Vitamin B6 μg	0.8	3.2
Vitamin B12 μg	1,5	6.0
Folacine μg	76	304
Vitamin C mg	23	92
Sodium g	0.4	1.6
Potassium g	0.7	2.8
Calcium g	0.4	1.6
Chloride g	0.6	2.4
Phosphor mg	400	1600
Magnesium mg	140	560
Iron mg	5.5	22.0
Zink mg	4.8	19.2

Blood samples were analyzed for HbA_1c_, fasting plasma glucose (FPG), cholesterol and triglycerides. Measurements were done at inclusion and after four weeks of diet. Change in blood pressure and weight was noted. Blood samples were also analyzed for electrolytes, blood cells, CRP and liver enzymes, see Table 3.

Statistical analysis was performed with Stata version 13.1. Skewness/Kurtosis test was used for assessment of distribution of data (parametric or non-parametric). Students t-test and Wilcoxon signed-rank test were used for parametric and non-parametric analysis of differences, respectively. P-value <0.05 was considered statistically significant.

## Results

**Figure 1:**
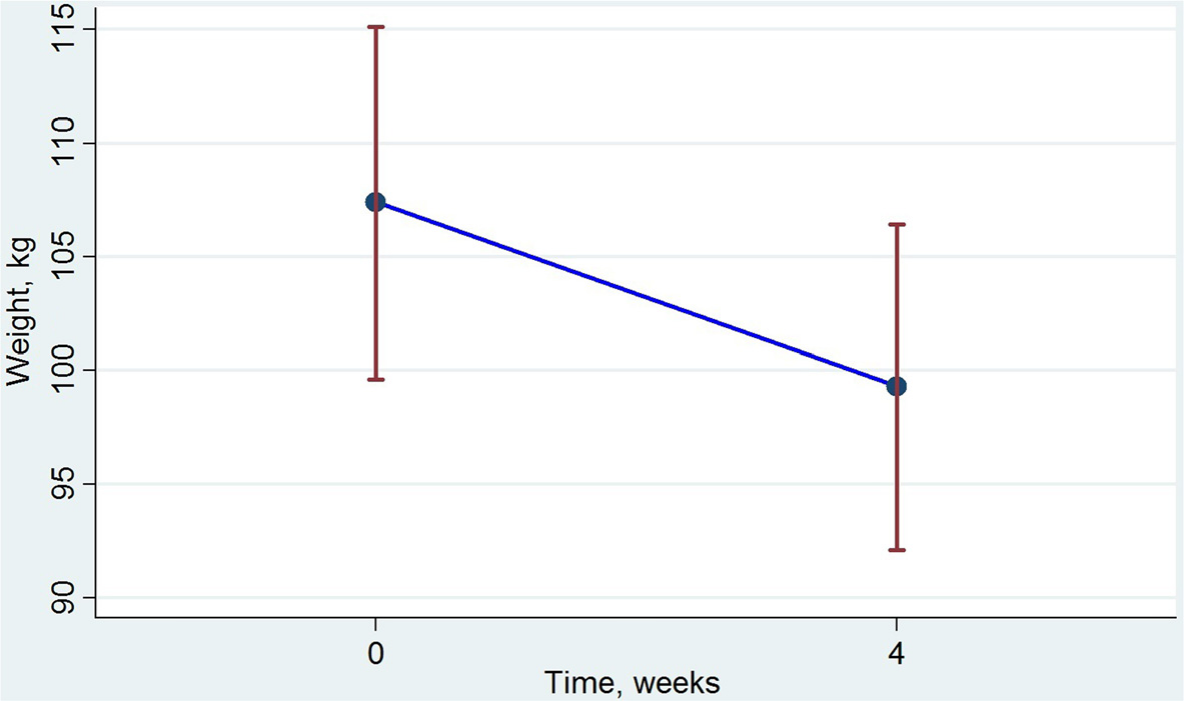
**Change in body weight with VLCD.** Mean weight (95% confidence interval), kg, before and after 4 weeks dietary treatment with fructose containing VLCD.

After four weeks of fructose containing VLCD all 25 subjects had mean weight loss of 8.2 kg as presented in Figure 1. Median excess weight (exceeding BMI 25) loss was 19.5% (inter quartile range 16.8-24.2). Changes in weight, blood pressure, lipids and FPG are presented in Table 4*.* All variables except diastolic blood pressure (DBP) were significantly reduced. DBP was significantly increased. It is noteworthy that at inclusion 15 subjects had elevated SBP (above 140 mm Hg). After diet 10 of these were improved but 5 subjects still had elevated SBP.

**Table 4:** Changes in metabolic variables; weight, blood pressure, glucose and lipids

***All subjects***				
***Variable***	***Inclusion***	***After diet, four weeks***	***P***	***N***
Weight (kg)*	107.4 (99.6-115.1)	99.3 (92.1-106.4)	0.001	25
BMI (kg/m^2^)*	39.8 (38.0-41.6)	36.8 (35.1-38.6)	0.001	25
Excess weight*	40.2 (34.5-45.9)	32.1 (26.8-37.4)	0.001	25
SBP (mmHg)^§^	150 (140–150)	135 (125–140)	0.005	25
DBP (mmHg)^§^	85 (80–90)	90 (85–95)	0.04	25
Glucose (mmol/l)^§^	5.6 (5.3-6.8)	4.8 (4.6-5.2)	0.001	24
HbA_1c_ (mmol/mol)^§^	39 (37–44)	37 (35–43)	0.001	23
Cholesterol (mmol/l)^§^	5.4 (4.9-6.2)	4.4 (4.1-4.9)	0.001	22
Triglycerides (mmol/l)^§^	1.6 (1.1-2.2)	1.2 (1.1-1.6)	0.006	21
***Subjects with type 2 diabetes mellitus***				
***Variable***	***Inclusion***	***After diet, four weeks***	***P***	***N***
Weight (kg)*	119.2 (99.3-139.1)	110.3 (91.5-129.1)	0.02	7
Glucose (mmol/l)^§^	7.5 (7.0-12.5)	6.0 (4.8-6.8	0.02	7
HbA_1c_ (mmol/mol)^§^	47 (44–66)	46 (45–59)	0.11	7

SBP = Systolic blood pressure. DBP = Diastolic blood pressure. *Presented as mean (95% confidence interval). ^§^Presented as median (inter quartile range). P-value denotes the level of statistical significance in the difference between value at inclusion and after diet.

**Figure 2:**
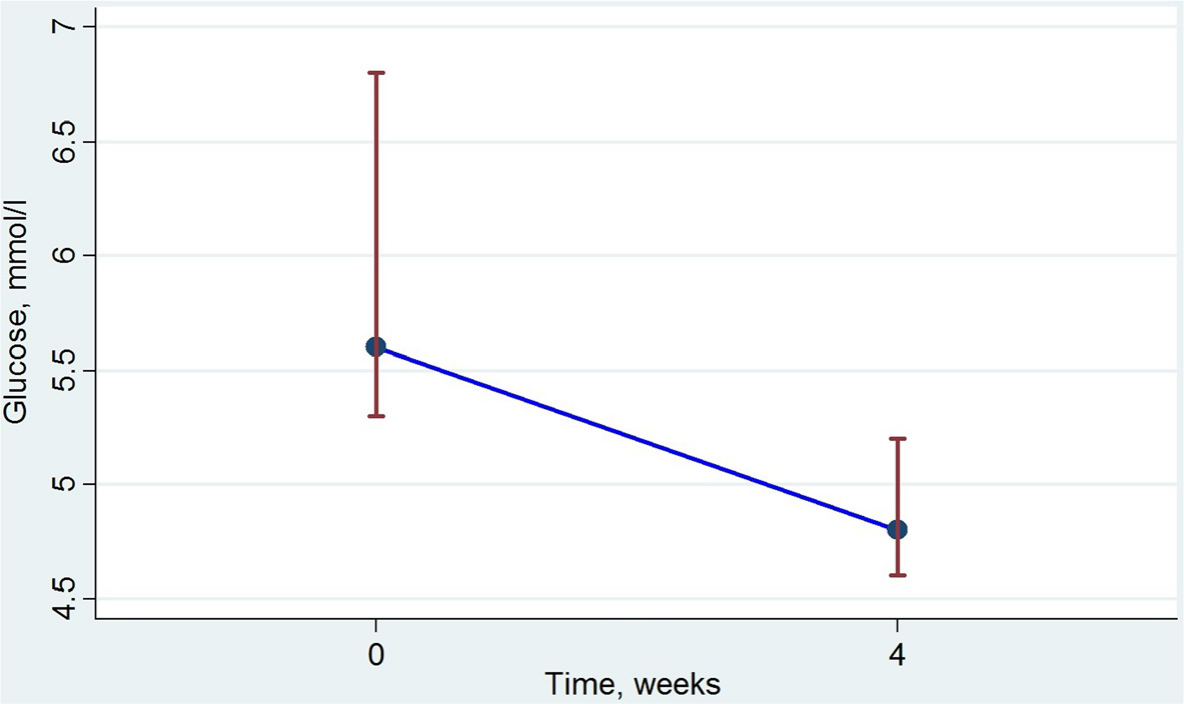
**Change in fasting glucose with VLCD.** Median fasting plasma glucose level (inter quartile range), mmol/l, before and after 4 weeks dietary treatment with fructose containing VLCD.

**Figure 3:**
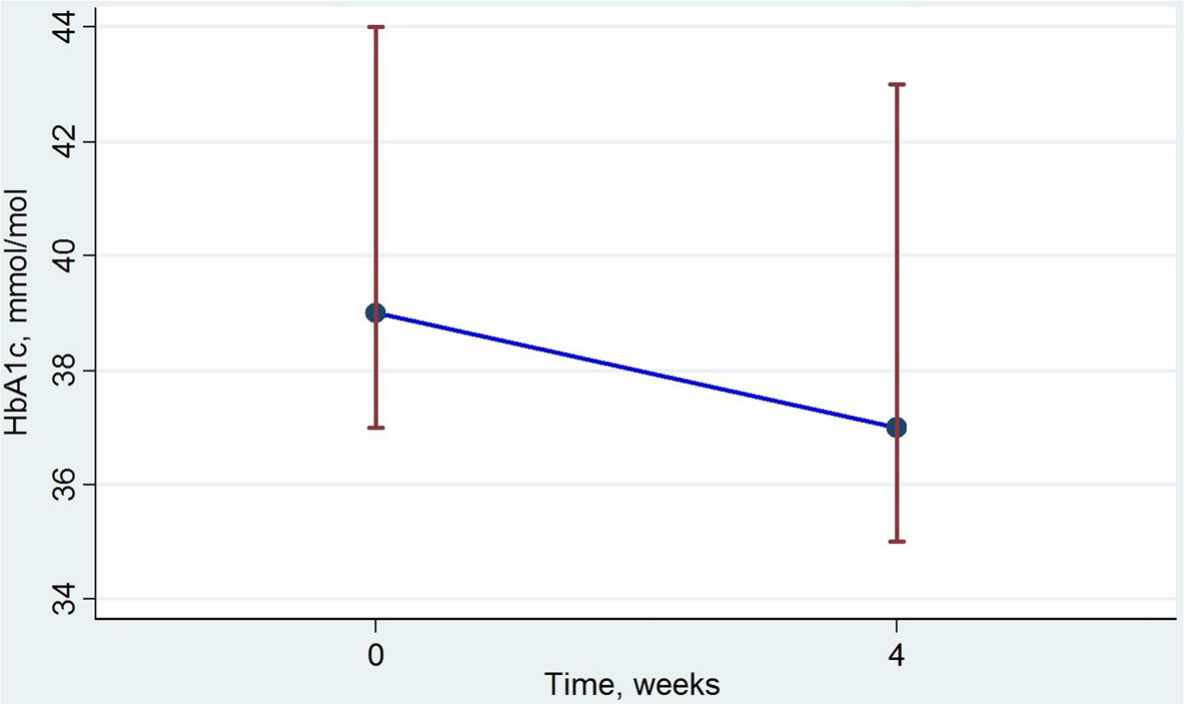
**Change in HbA1c with VLCD.** Median HbA_1c_ level (inter quartile range), mmol/mol, before and after 4 weeks dietary treatment with fructose containing VLCD.

Five of the seven subjects with T2DM had FPG above 7.0 mmol/l at inclusion. One subject without known T2DM also presented with FPG above 7.0 mmol/l. Three subjects with T2DM presented with HbA_1c_ levels above 52 mmol/mol at inclusion, indicating insufficient treatment T2DM control. One of these improved during treatment with VLCD, and reached normal level of HbA_1c_. The only subject who persisted with FPG above 7.0 mmol/l after four weeks of diet was the patient with the highest FPG at inclusion, who also had a persisting elevated HbA_1c_ value. Change in FPG and HbA_1c_ levels are presented in Figure 2 and Figure 3, respectively. Change in weight, FPG and HbA_1c_ levels for the diabetic subjects are also presented separately in Table 4.

Triglyceride levels above 1.7 mmol/l were present in 11 subjects at inclusion, and improved in 6 of these after treatment. Similar results were found regarding cholesterol. Seventeen subjects had cholesterol above 5.0 mmol/l at inclusion and 12 improved after dietary treatment.

Blood chemistry of electrolytes and blood cell count are displayed in Table 5*.* There were only minor or no changes indicating no clinically significant adverse effects of the diet. Liver enzymes AST and ALT were significantly increased, but still well within normal range and the changes were minor.

**Table 5:** Laboratory variables

***Variable***	***Inclusion***	***After diet, four weeks***	***P***	***N***
CRP (mg/l)*	5.0 (4.9-8.0)	<5	0.04	25
Hemoglobin (g/l)	135 (132–140)	138 (132.5-143.5)	0.01	24
Trombocytes (10^9^/l)	325 (244–389)	316 (203–357)	0.01	24
White blood cells (10^9^/l)	6.5 (5.9-7.8)	6.8 (5.95-7.6)	0.71	24
Mean cellular volume (fl)	88 (86–91)	88 (85.5-90.5)	0.12	24
Sodium (mmol/l)	140 (139–141)	139 (139–141)	0.91	25
Potassium (mmol/l)	4.3 (4.1-4.5)	4.3 (4.1-4.4)	0.04	21
Calcium (mmol/l)	2.37 (2.32-2.41)	2.42 (2.36-2.47)	0.06	25
Creatinine (μmol/l)	61 (53–67)	76 (53–75)	0.19	23
Bilirubin (μmol/l)	8 (7–10)	9 (8–12)	0.03	25
ALT (μkat/l)	0.48 (0.37-0.64)	0.53 (0.39-0.98)	0.01	25
AST (μkat/l)	0.35 (0.30-0.44)	0.42 (0.34-0.62)	0.001	24
ALP (μkat/l)	1.36 (1.11-1.48)	1.26 (0.91-1.39)	0.02	25

Changes in non-metabolic blood chemistry values. Presented as median (inter quartile range). P-value denotes the level of statistical significance in the difference between value at inclusion and after diet. *Low-sensitive CRP.

## Discussion

Four weeks of the fructose containing VLCD Slanka led to improved metabolic control. Overweight was reduced with almost 20%. FPG, HbA_1c_, triglycerides and cholesterol levels were significantly reduced. Blood pressure was improved.

Liver enzymes were slightly elevated. There is evidence that isocaloric exchange of fructose for other carbohydrates does not induce non-alcoholic fatty liver disease in otherwise healthy individuals. Diets containing high doses of fructose (more than 100 g per day) may induce changes associated with non-alcoholic fatty liver disease [11]. The small changes in liver enzymes, well within normal range, in the context of a small dose of fructose (20 g per day) leads to the conclusion that this change is not a cause for concern during a 4 week treatment.

Although our results clearly indicate improved metabolic control we can draw no certain conclusion as to how the fructose component affected the metabolism. Our study lack a control group and we therefore have to compare with previously published results, summarized in Table 6.

**Table 6:** Literature review of demographics and FPG

***Study***	***N (♀/♂)***	***Age***	***BMI***	***FPG at inclusion***	***FPG after diet***	***Country***	***Comments***
Present study	25 (23/2)	48.8 (1,66)^‡^	39.8 (0.9)^‡^	5.6 (5.3-6.8)^#^	4.8 (4.6-5.2)^#^	Sweden 2013	7 subjects with T2DM. 4 weeks diet.
Case	125 (?/?)	48.4 (10.4)*	40.7 (9.7)*	6.4 (2.1)*	5.4 (1.2)*	U.S. 2002	Metabolic syndrome. 4 weeks diet.
Wikstrand [12]	46 (?/?)	46 (9)*	35.4 (3,4)*	5.2 (0.9)*	4.8 (0.5)*	Sweden 2010	Prevalence of T2DM unknown. 12 weeks diet.
- Group A							
Wikstrand [12]	48 (?/?)	48 (9)*	36.1 (3.7)*	5.3 (2.2)*	4.7 (0.6)*		
- Group B							
Yunjuan [14]	53 (?/?)	?	32.6 (0.6)^‡^	5.3 (0.1)^‡^	5.2 (0.1)^‡^	China 2013	Healthy, no T2DM. 8 weeks diet.
Jackness [15]	11 (7/4)	44.6 (3.0)^‡^	43.2 (2.3)^‡^	9.9 (1.2)^‡^	6.9 (1.2)^‡^	U.S. 2013	All T2DM. 3 weeks diet.
- RYGP							
Jackness [15]	14 (8/6)	51.9 (2.0)^‡^	39.2 (1.0)^‡^	10.2 (0.7)^‡^	6.1 (0.3)^‡^		
- VLCD							

Literature review demographics and FPG in different studies. Search was performed using http://www.pubmed.gov with “VLCD” in combination with either “glucose”, “aspartame” or “fructose” as search text. The review was arbitrarily limited to articles published the last 20 years, between 1993 and 2013 and only to include full-text publications. Abstracts were reviewed in order to find results related to the aim of this study; primary outcome FPG levels after VLCD. Fasting serum glucose was treated equal to fasting plasma glucose [11]. All studies except Jackness et al. are observational regarding VLCD. Both groups in Wikstrand et al. received similar VLCD-regime. *Presented as mean (standard deviation). ^‡^Presented as mean (standard error). ^#^Presented as median (inter quartile range).

An earlier Swedish trial [12] by Wikstrand et al. investigating an adjunctive 9 months abdominal corset (ie an circumferential abdominal restrain device) treatment versus control after 3 months of VLCD in obese subjects has shown similar results of the dietary treatment as we find in our study. The VLCD (Nutrilett, Cederroth International AB) contains fructose, Acesulfame K and aspartame. The subjects in this study were of same population and age as ours, but had somewhat less overweight. It is interesting to note that the metabolic improvement partially persists 9 months after the diet was finished and is similar to that which we saw immediately after 4 weeks of diet in our study.

Case et al. [13] reports a similar reduction of FGP in an observational consecutive VLCD program in the US including 125 subjects with metabolic syndrome. The VLCD (Nutrimed-Plus, Robard Corporation, Mount Laurel, NJ, USA) used in the study contains approximately 600–800 kcal per day and 30–40 g carbohydrate per day. VLCD in our study contains approximately 70 g carbohydrate per day.

Gu et al. performed an observational study [14] in a Chinese population including 53 overweight or obese (BMI > 28) but otherwise healthy subjects. The subjects consumed VLCD with less than 800 kcal per day and less than 20 g carbohydrate per day for 8 weeks. As seen in Table 6 our diet with a higher amount of carbohydrate led to a more favorable change in FGP level despite shorter treatment and presence of significant comorbidities. The difference between studies can partially be explained by the absence of T2DM – and thus normal FGPD at inclusion – in the Chinese group.

Jackness et al. [15] compared two groups of obese subjects with T2DM in the US. One group underwent bariatric surgery with Roux-en-Y gastric bypass (RYGBP) and the other had VLCD for three weeks. Both groups had high levels of HbA_1c_ at inclusion (mean 66 and 69 mmol/mol respectively). Insulin sensitivity, β-cell function and glucose tolerance were measured. The authors conclude that the early beneficial effects after RYGP probably are caused by caloric restriction. Results are strengthened by the Netherlands study of Lips et al. [16]. Our study group contains mainly subjects without T2DM. HbA1c at inclusion was median 39 mmol/mol. Therefore, as expected, the reduction in FPG is not as explicit in our results.

It has been shown that obese subjects with T2DM started on VLCD, in whom all glucose lowering medication has been discontinued, some subjects respond with reduction of FPG almost immediately, while others did not respond at all. This change in FPG is probably dependent on the capacity of β-cells to secrete insulin, and is independent of weight change [17]. The same research group has also shown that the positive metabolic effects of 30 day VLCD leads to a sustained improvement in metabolic control up to 18 months follow-up [18].

Although VLCD and other calorie restricted diets have been widely used for many years, there is still scarce of large randomized clinical trials. The effectiveness of a diet is often improved by combination with physical exercise and behavioral interventions [19]. In summary, there is little evidence available to support the extended use of VLCD for weight reduction in obese adults. Little is known of long-term health outcome and quality of life [20]. This is in contrast to the long-term effects of bariatric surgery, which has been studied in detail with known positive effects [3].

We do not find any indicator of metabolic adverse effects resulting from the replacement of aspartame with fructose. Our results with fructose containing VLCD seem to be equivalent to earlier published results from VLCD with artificial sweetener in short-term outcome. The addition of fructose seems to be of lesser importance than severity of hyperglycemia at start of diet. Although there is no strong evidence against use of artificial sweeteners as food additives there are still subjects whom out of personal believe wish to avoid consuming artificial additives. In obese patients preparing for bariatric surgery with VLCD there seems to be no negative metabolic effects regarding blood samples and laboratory analysis measured in this study. Based on this limited observational study we do not find any contraindications for VLCD with fructose instead of artificial sweetener before bariatric surgery.

## Abbreviations

BMI: Body mass index; VLCD: Very low calorie diet; T2DM: Type 2 diabetes mellitus; FPG: Fasting plasma glucose; DBP: Diastolic blood pressure; SBP: Systolic blood pressure; RYGBP: Roux-en-Y gastric bypass.

## Competing interests

EN and HF received grant from Slanka Sverige AB in the form of the actual diet for the study as well as financial support for the article processing charge for online publishing. Slanka Sverige AB did not have any part in the initiation or design of the study. EN and HF received grant from Mina Medical AB for a research project regarding a novel endoscopic device for treatment of obesity.

## Authors’ contributions

EN participated in the design of the study, performed the statistical analysis, and drafted the manuscript. HF conceived of the study, and participated in its design and coordination and helped to perform the statistical analysis and draft the manuscript. Both authors had read and approved the final manuscript.
